# Creating a population-wide Desirable Health Service Utilisation Indicator using linked health and administrative data

**DOI:** 10.23889/ijpds.v9i5.2527

**Published:** 2024-09-18

**Authors:** Lucy Robinson

**Affiliations:** 1 Welsh Government; 2 ADR Wales

There are separate data collections across education phases in Wales (UK). Each use a different unique learner identifier. The matched education dataset project used advanced data linking methods to produce a set of pseudo identifiers for each learner that can be matched back to the original datasets to undertake specific, de-identified analysis.

The first phase of the project involved data cleaning, preparation, and the creation of new derived variables. The second phase established the linking methodology, developing and advanced data linking techniques and algorithms including frequency matching and phonetic string comparators. At each stage of the project the data linking was sequential, ranging from exact to more fuzzy matching. During the code development the approach was uniquely tailored to each data set and constantly fine-tuned to ensure the highest possible match rate while reducing potential for false matches.

The resulting linked education data sets are used in a number of ways for statistical and research purposes to support the formation of evidence-based policies. This has included research into raising the compulsory education age and the evaluation of learner journeys during the pandemic.

Robust linked data facilitates analysis examining the progression of learners through the education system in Wales. There is a broad scope of future analysis planned and the matched education outputs will be used extensively in the evaluation of learner journeys post pandemic to inform Welsh Government policy.

The matched education dataset project involved researching and upskilling in data linkage methodology, we hope to disseminate the lessons learned widely.

